# A Study on Internal Defects of PREP Metallic Powders by Using X-ray Computed Tomography

**DOI:** 10.3390/ma14051177

**Published:** 2021-03-03

**Authors:** Yan Nie, Junjie Tang, Junfei Huang, Shu Yu, Yunping Li

**Affiliations:** 1 State Key Lab for Powder Metallurgy, Central South University, Changsha 410083, China; nieyan@csu.edu.cn (Y.N.); tangjunjie@csu.edu.cn (J.T.); 2 Yuanmeng Precision Technology (Shenzhen) Institute, Shenzhen 518110, China; 3 Shimadzu (China) Co., Ltd. Shenzhen Branch, Shenzhen 518000, China; sszhjf@shimadzu.com.cn

**Keywords:** plasma rotating electrode process (PREP), powder, internal defects, synchrotron X-ray computed tomography

## Abstract

In this study, the distribution, proportion and characteristics of internal defects in three kinds of powders of Ti-6Al-4V, 316-steel and Co-29Cr-6Mo alloys, produced by the plasma rotating electrode process (PREP) at various rotation speeds, are characterized by using both scanning electron microscopy (SEM) and synchrotron X-ray computed tomography (CT). The results show that in the powder of a given alloy, internal pores are formed more easily in coarse particles than in fine powder during PREP. The proportion of powder with pores can be reduced by appropriately increasing the rotation speed. In addition, the composition of an alloy has a great influence on the defect formation.

## 1. Introduction

Additive manufacturing (AM) has attracted increasing attention due to its distinctive advantages such as design freedom, short manufacturing cycles, and simple manufacturing processes [1,2,3]. However, pore defects in additive manufactured products exhibit huge hidden dangers, which could significantly deteriorate both the mechanical and fatigue properties of the AM products [4,5]. Many investigations [4,5,6,7,8] have reported that most pores in additive manufacturing products originate from raw powders, and the pore defects in raw powders cannot be eliminated completely in the subsequent processing, such as with hot isostatic pressing (HIP). The characteristics of a powder strongly depend on the powder manufacturing technology.

Both PREP and gas atomization (GA) are the common methods in manufacturing suitable powders used in the AM process with both high sphericity and high flowability [9,10,11]. Although some pore defects were observed in the powder prepared by the two processes, the characteristics of the pores are quite different [6]. In the last decades, many studies have been conducted on the pore characteristics in powders of different alloys [7,8,12,13,14,15,16,17,18]. Chen [6] found that the number and size of pores as well as the porosity and argon gas trapped in Ti-6Al-4V powders increase significantly with increasing particle size. Susan [12] produced stainless steel powder by GA and found that the interlayer porosity increased with increased powder porosity. The pore characteristics in powders prepared by two processes were also compared. Chen [7] reported that GA powders demonstrate higher porosity than the corresponding PREP powders. However, there have been relatively few studies on the internal pores of PREP powder, in particular, the effects of PREP process parameters, such as rotation speed and alloy type, on the pore characteristics have not been discussed in detail.

Scanning electron microscopy (SEM) and optical microscopy (OM) are two common characterization methods used to characterize the internal pores of powder [10,19,20]. However, both techniques have some shortcomings in analyzing the pore characteristics. One of the major drawbacks is that observation of a cross-section of powder by SEM or OM requires complex pretreatment such as grinding and polishing, which greatly influences the final results. Furthermore, the results obtained by SEM or OM only reflect the information from the two-dimensional plane inside the powder. With this in mind, High-resolution synchrotron X-ray computed tomography (CT) was used to quantitatively analyze the information of pores in powders [21,22,23,24]. The distribution, pore spatial morphology and fraction of pores can be observed clearly in the powder by 3D reconstructing the entire morphology of powders.

Therefore, in this paper, both SEM and CT were used to characterize the inner microstructure of PREP powder, and the characteristics of the pores were observed from the two-dimensional plane and three-dimensional space respectively. The distribution, spatial shape, size of the internal pores of different alloy powders by PREP are characterized in detail by combining SEM and synchrotron X-ray CT. The purpose of this study is to investigate the effects of rotation speed, particle size and alloy type on pore characteristics systematically.

## 2. Experimental

Three alloy ingots (Ti-6Al-4V (TC4), Co-29Cr-6Mo (CCM), 316-steel (316L)) were processed into cylindrical bars with Φ75 mm diameter by vacuum induction melting (VIM). The detailed chemical compositions of the three alloys have been given in previous studies [25]. Three cylindrical bars were processed into powders by PREP (SL-01, Sailong Metal, Xi’an, China) at four different rotation speeds: 8000; 10,000; 12,000 and 14,000 RPM. The powder of a given alloy and a given rotation speed is sieved into three particle size ranges or three batches, separately. Laser particle size analyzer (LPSA) (Mastersizer 3000E, Malvern Panalytical, Worcestershire, UK) was used to analyze the particle size distribution of powder. After the PREP process, there was no significant difference in the main elemental composition of alloys except that the O content was increased by lower than 200 ppm in all alloy powders. The pore characteristics of powders were characterized by scanning electron microscope (FEI Quanta 650, FEI, Tokyo, Japan) and Micro-computed tomography (Micro-CT) (Inspe Xio SMX-225 CT FPD HR, Shimadzu Co., Ltd., Tokyo, Japan), respectively. To clearly observe the cross-sectional morphology of powder by SEM, each powder is mounted in conductive Mosaic powder, respectively, and then polished by using 0.3 μm alumina suspension. In order to observe the spatial structure of pore, the powders were analyzed qualitatively by Micro-CT at an accelerating voltage of 225 kV and a resolution of 4 μm. Three-dimensional (3D) structure was reconstructed by using the VG Studio MAX software (v3.2). To quantitatively count the pores in powder, three random areas were selected, and then the mean number fraction of powder with pores was obtained.

## 3. Results

**Figure 1:**
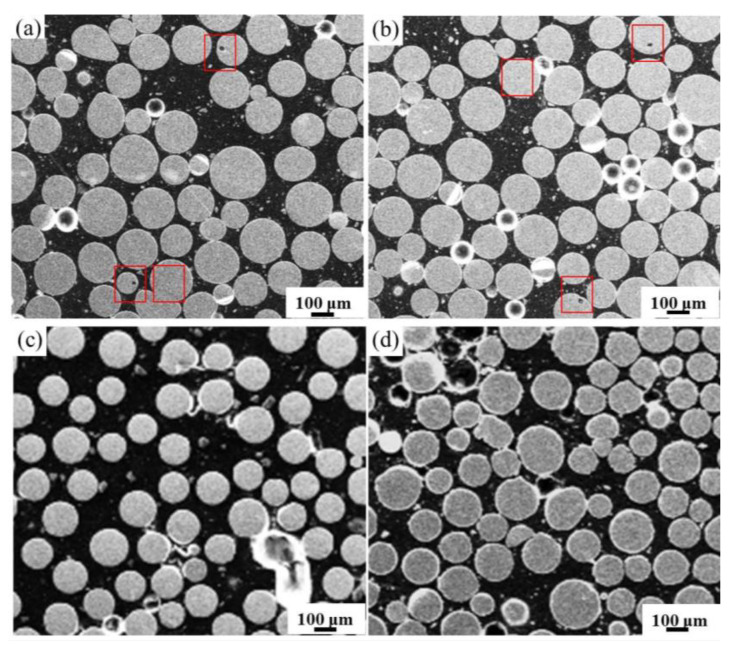
Cross-sectional morphology of Ti-6Al-4V alloy powders at (**a**) 8000; (**b**) 10,000; (**c**) 12,000 and (**d**) 14,000 RPM.

Figure 1 shows the cross-sectional morphology of Ti-6Al-4V powder at four rotation speeds by SEM. It can be figured from the cross-section morphology that the average particle size of the powder gradually decreases with the increasing rotation speed from 8000 to 14,000 RPM, and some satellite powder and irregular powder can be observed. The results are consistent with our previous results [25]. Furthermore, some pores can be observed at 8000 and 10,000 RPM (Figure 1a,b), while pores can hardly be observed at 12,000 and 14,000 RPM (Figure 1c,d). The pores are mostly located at the edge and center of the powder.

**Figure 2:**
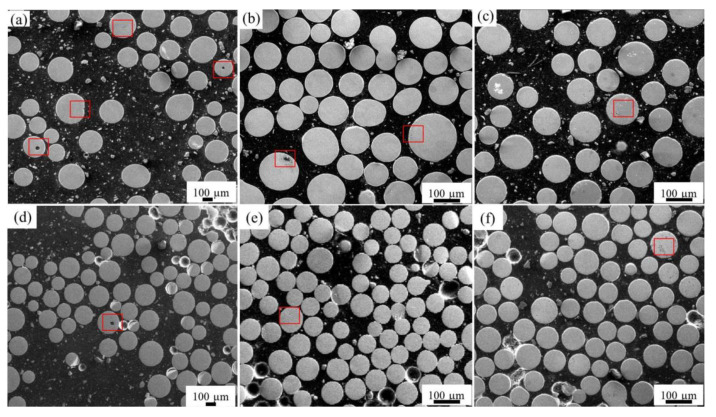
Cross-sectional morphology of (**a**) Ti-6Al-4V, (**b**) Co-Cr-Mo, (**c**) 316-steel at 8000 RPM, (**d**) Ti-6Al-4V, (**e**) Co-Cr-Mo, (**f**) 316-steel at 12,000 RPM.

Figure 2 shows the cross-sectional morphologies of the three alloy powders at 8000 and 12,000 RPM respectively. Similarly, some pores can also be observed on the cross-sectional morphology of Co-Cr-Mo and 316L alloy powder (Figure 2b,c). An obvious difference from the Ti-6Al-4V alloy is that the pore size of Co-Cr-Mo and 316L alloy powders is very small, and there are many small pores in the cross-section of the powder. From the results observed by SEM, the proportion of powder with pores in three alloy powders does not change significantly with the increase in rotation speed, but the amount of powder with pores is slightly higher at a low rotation speed.

**Figure 3:**
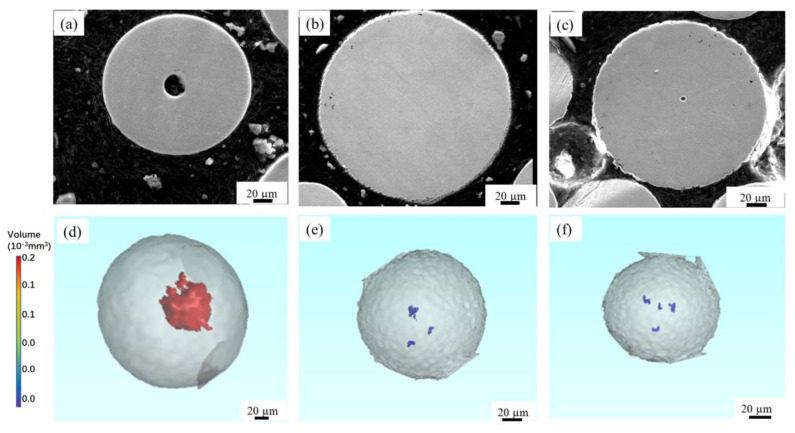
Cross-sectional morphology of the alloy powders by scanning electron microscopy (SEM) at 8000 RPM (**a**) Ti-6Al-4V, (**b**) Co-Cr-Mo, (**c**) 316-Steel [25]; 3D reconstructed computed tomography (CT) images of (**d**) Ti-6Al-4V, (**e**) Co-Cr-Mo, (**f**) 316L.

In order to clearly observe the morphology of pores, three alloy powders were observed by SEM with a high magnification at 8000 RPM, as shown in Figure 3a–c. First, the pore size is about 20 μm in the Ti-6Al-4V particle of about 200 μm, which is the largest among the three alloy powders. However, the pores in both Co-Cr-Mo and 316L powders are extremely small (about 2–5 μm), although the particle size of the two alloys are comparable to that of Ti-6Al-4V alloy. Second, it can be seen clearly that there are multiple pores inside the Co-Cr-Mo and 316L alloy powders, which are distributed mostly near the edge of the powder. As mentioned before, the observations by SEM have some technical limitations, as the spatial information of pores cannot be reflected on a two-dimensional plane. Therefore, 3D reconstruction images of the powders were obtained by CT, as shown in Figure 3d–f. The colored objects inside the powder represent the pore, and the different colors represent the volume of the pore. From the reconstructed images (Figure 3d–f), it can be found that the morphology, volume and number of pores in three alloy powders can be observed more intuitively. In terms of the pore volume, the volume of pores in Ti-6Al-4V alloy powder is large, while that in Co-Cr-Mo and 316L alloy powders is very small. In terms of number of pores, the number of pores in Ti-6Al-4V alloy powder is almost only one, while in Co-Cr-Mo and 316L alloy powders it is mostly more than two. The results are basically consistent with those observed by SEM. In addition, it is found that the pores in Ti-6Al-4V alloy powders exhibits a quasi-spherical shape, while the pores in Co-Cr-Mo and 316L alloy powders exhibit an irregular shape.

**Figure 4:**
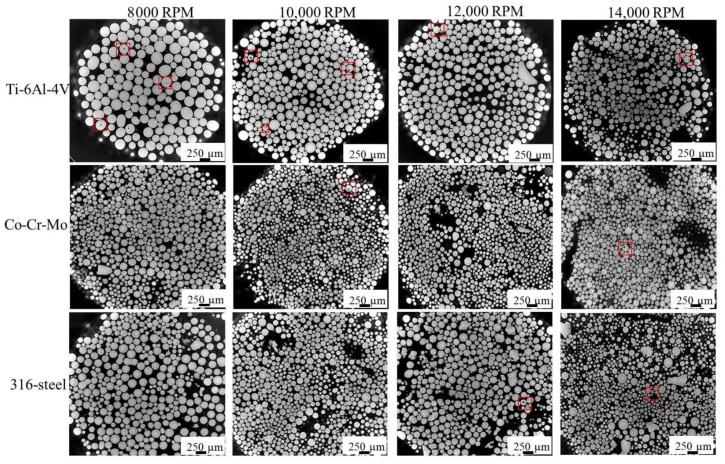
Synchrotron X-ray CT cross-sectional morphology of 3 alloy powders by at various rotation speeds [25].

Figure 4 shows the cross-sectional morphology of three alloy powders by synchrotron X-ray CT. The following conclusions can be drawn from the CT results. First, the particle size at low rotation speed is generally coarser than the powder at high rotation speed, which has been analyzed in a previous study [10]. Second, many pores can be observed in Ti-6Al-4V alloy powders, while only a small amount of pores exist in Co-Cr-Mo and 316L alloy powders. The results are consistent with those observed by SEM. Meanwhile, it is found that pores can be observed at high rotation speeds (12,000 and 14,000 RPM) by CT, which is not observed by SEM. The fraction of powder with pores at a low rotation speed is obviously higher for Ti-6Al-4V powder, while no obvious change is found in the other two kinds of alloy. Furthermore, pores are frequently found in coarse particles, while fewer pores are observed in fine particles.

**Figure 5:**
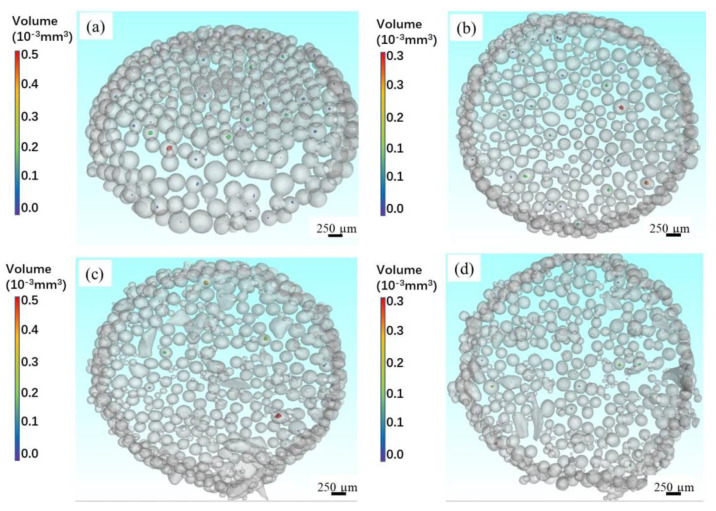
The 3D reconstructed CT of Ti-6Al-4V powders at (**a**) 8000 RPM, (**b**) 10,000 RPM, (**c**) 12,000 RPM, (**d**) 14,000 RPM.

Whether the pores in powder are observed by SEM or CT scanning, the observation of the cross-sectional morphology is only for the two-dimensional plane with a thickness of a few microns inside the powder. Therefore, some unexplored areas in powder could be ignored and the three-dimensional information of the pore also cannot be fully reflected. Figure 5 shows the 3D reconstructed CT images of Ti-6Al-4V powders at four different rotation speeds. The PREPed powder has a high sphericity, but irregular powder can also be observed. Furthermore, the distribution, spatial morphology and geometry of pores can be clearly observed from the 3D reconstructed CT images. The following important information can be obtained from the 3D reconstructed CT images. First, it is further verified that there are more powders with pores at the low rotation speed. Second, for Ti-6Al-4V alloy, most powders with pores have only one pore inside. Third, the volume of pores in each powder is different.

**Figure 6:**
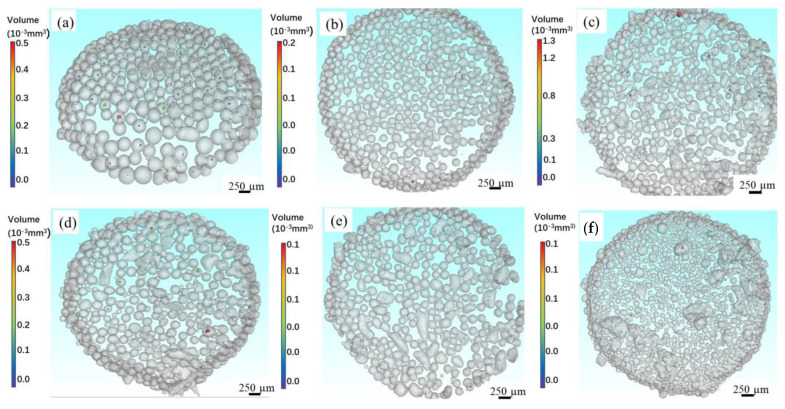
The 3D reconstructed CT of (**a**) Ti-6Al-4V, (**b**) Co-Cr-Mo, (**c**) 316L at 8000 RPM; as well as (**d**) Ti-6Al-4V, (**e**) Co-Cr-Mo, (**f**) 316-steel at 12,000 RPM.

**Figure 7:**
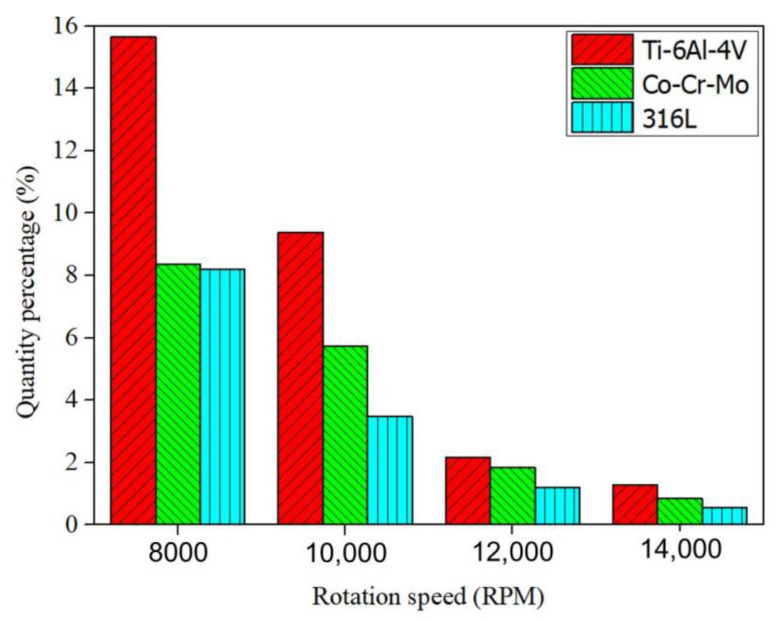
Quantify percentage of powder with pores in the plasma rotating electrode process (PREP) powders as a function of rotation speed.

Figure 6 shows the 3D reconstructed CT images of three alloy powders at 8000 and 12,000 RPM, respectively. Similarly, many pores can also be observed in Co-Cr-Mo and 316L alloy powders, and the proportion of powder with pores in as-atomized powder at 8000 RPM is also higher than that at 12,000 RPM. However, the difference with Ti-6Al-4V alloy powder is that multiple pores are observed in a powder for Co-Cr-Mo and 316L alloy powders. Especially in irregular powders, there seem to be more pores. In addition, compared with Ti-6Al-4V alloy powder, the volume of pore in Co-Cr-Mo and 316L alloy powders is generally smaller. The quantity percentage of powder with pores in as-atomized powders at each rotation speed is quantitatively analyzed according to the data obtained from CT images, as shown in Figure 7. For a certain alloy, the quantity percentage of a pore decreases with increasing rotation speed. Especially at low rotation speeds (8000 and 10,000 RPM), the quantity percentage of powder with pores is especially large. For example, the quantity percentage of powder with pores in Ti-6Al-4V powder is 15.64% at 8000 RPM, while it is 1.78% at 14,000 RPM. In addition, the quantity percentage of powder with pores in Ti-6Al-4V powder is significantly higher than that of both Co-Cr-Mo and 316L alloy powders. The quantity percentage of powder with pores in Ti-6Al-4V powder is 15.64% at 8000 RPM, while it is 8.58% and 8.26% of Co-Cr-Mo and 316L alloy powders at 8000 RPM, respectively.

**Figure 8:**
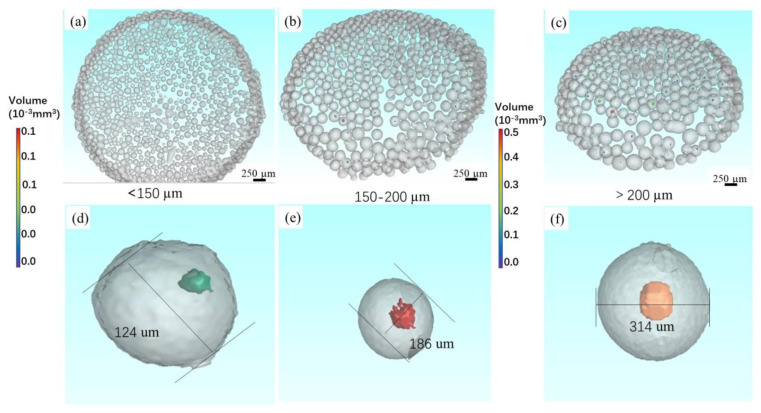
The 3D reconstructed CT of Ti-6Al-4V powders at 8000 RPM with different size ranges. (**a**) <150 µm, (**b**) 150–200 µm, (**c**) >200 µm and the typical pore morphology for particle in the particle range of (**d**) <150 µm, (**e**) 150–200 µm and (**f**) >200 µm.

**Figure 9:**
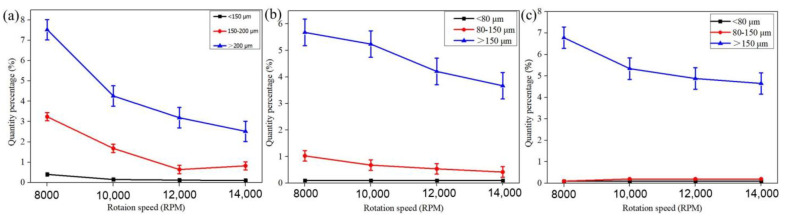
Quantity percentage of powder with pores in PREP powders as a function of particle size range and rotation speed. (**a**) Ti-6Al-4V, (**b**) Co-Cr-Mo, (**c**) 316L.

Figure 8 shows the 3D reconstructed CT of Ti-6Al-4V powders at 8000 RPM with three different size ranges. Among the powders with particle size less than 150 µm, the number of powders with pores inside is few (Figure 8a). As the particle size increases to 150–200 µm (Figure 8b), it can be seen that powders with pores inside increase gradually. Especially in powders larger than 200 µm (Figure 8c), the quantity of powders with pores is particularly large. Quantitative analysis of quantity percentage for three powders with different size ranges is performed according to the data achieved by CT images, as shown in Figure 9. Whether it is Ti-6Al-4V, Co-Cr-Mo or 316L powder, the quantity percentage of pores in the powders with a larger particle size is obviously more than in powders with a small particle size. For example, the quantity percentage of powder with pores in Ti-6Al-4V powder with a particle size larger than 200 µm is 7.58%, while it is 3.24% and 0.45% for powders with particle sizes between 150 and 200 µm and less than 150 µm, respectively. Meanwhile, pores are almost not observed in Co-Cr-Mo and 316L alloy powders with particle size ranges of less than 80 µm. In addition, one interesting aspect is that the size of pores gradually increases with the increase in particle size (Figure 8d–f). It can be observed clearly that the volume of pores in the powders with a particle size of 314 µm (Figure 8f) is larger than that in the powder with a particle size of 186 µm (Figure 8e), and even the volume of pores in the powder with a particle size of 124 µm (Figure 8f) is smaller.

## 4. Discussion

### 4.1. Effect of Particle Size on Pore Characteristics

During the atomization of PREP, the formation of pores in the powder is mainly due to the following reasons. First, the difference from the GA method is that the spheroidization and solidification process of PREP droplets does not occur simultaneously. The molten metallic droplets are thrown from the end surface of electrode bar, and then solidified into particles during flight cooling in argon. Therefore, the molten metal droplet may enwrap some gas inside itself during flight. Second, some of the pores in powder may also be formed by the transfer of gas from the surface to the inner region of the droplet under the action of thermal gradient [6,15,16]. Finally, it is well known that the plasma gun blowing Ar gas is mounted facing directly towards the surface end of the rotating rod; during heating on the end surface of rod, the blowing gas is extremely easily trapped by the melted surface, which finally forms into droplets together with the trapped gas. This is consistent with the results in the highest number fraction of pores in Ti-6Al-4V alloy powder, since gas is much more easily trapped by the liquid Ti-6Al-4V alloy with the lowest surface tension.

It can be figured from Figure 8 and Figure 9 that particle size plays a key role in the size and quantity percentage of pores. Pores are more likely to form in larger particles, which may be related to surface tension. As mentioned in previous studies, small metal droplets have a faster cooling rate than big droplets, and so small metal droplets have a higher surface tension [6,26,27]. Another reason is that the area where the droplet breaks is exactly the area where the pores form, many small droplets are formed by further fragmentation of large droplets. Based on the above reasons, larger particles (≥200 µm) exhibit a higher possibility of forming pores than small particles. In addition, it is also found that powders with pores of large sizes have larger pores than Ti-6Al-4V, as shown in Figure 8d–f). Larger particles have large internal spaces and lower surface tension, in which argon is more easily adsorbed and stored compared to the smaller particles at a given rotation speed.

### 4.2. Effect of Rotation Speed and Alloy Type on Pore Characteristics

Rotation speed of the electrode bar and alloy type also play a key role in formation of powder with pores. The results in Figure 9 show that the quantity percentage of powder with pores is significantly less at a high rotation speed. There are two main reasons: first, the proportion of coarse powder is large at a low rotation speed, and pores are easily formed in coarse powders. Second, the linear velocity of the droplets is fast when droplets are thrown out during rotation because the rotation speed of the electrode bar is too fast, and the droplet have solidified into particles before the argon is entrapped in droplet.

In terms of the amount of powder with pores, the quantity percentage of Ti-6Al-4V powder with pores is the highest among the three alloys for a given rotation speed, and the quantity percentage of powder with pores in Co-Cr-Mo powder is about the same as that in 316L powder (Figure 7). The reason for the difference in the number proportions of powder with pores is mainly attributed to the surface tension and density of the alloy. Under the ligament disintegration (LD) mechanism, the mean particle size can be calculated by [28,29]

(1) $$D50=\frac{2.0}{\omega }\sqrt{\frac{\gamma }{\rho D}}$$

where γ is the surface tension, ω is rotation speed, ρ is density and *D* is electrode diameter. When the rotation speed and diameter of electrode bar are determined, the average particle size depends on the density and surface tension of the alloy. γ/ρ of Ti-6Al-4V, Co-Cr-Mo and 316L are 0.306, 0.229 and 0.219, respectively. Therefore, the average particle size of Ti-6Al-4V is largest among three alloys, and large particles are more likely to form powders with pores, which is one of the main reasons why the number of powders with pores in Ti-6Al-4V alloy is higher than that in other two alloys. In addition, it is known from the above analysis that it is harder to absorb argon in droplets with high surface tension. It can be seen that the surface tension of Ti-6Al-4V alloy is the lowest among the three alloys, which is the another reason why the number fraction of Ti-6Al-4V powder with pores is higher than that of the other alloys.

In terms of the number of pores, there is usually only a single pore in each powder for Ti-6Al-4V alloy (Figure 3d), while each powder with pores usually contains multiple pores for Co-Cr-Mo and 316L alloys. In terms of the volume of pores, the volume of pores in Ti-6Al-4V powder is much larger than that in Co-Cr-Mo and 316L alloys (Figure 3e,f). Similarly, the reason for the difference in pore characteristics is also attributed to the difference in the properties of the alloy itself. Ti-6Al-4V with low surface tension may have a higher affinity for argon than Co-Cr-Mo and 316L, which results in a molten metal droplet that more easily absorbs argon during solidification.

In spite of the aforementioned internal defects in PREP powder, PREP powder shows much lower defects and higher flowability compared to the traditional GA, implying that the PREP powder is more suitable to be used in the additive manufacturing process. However, since both the particle size and the internal defects of PREP powder are strongly dependent on the rotation speed, increasing the rotation speed in large scale seems much more imperative in future.

## 5. Conclusions

In this paper, the pore characteristics such as distribution, proportion and size of the internal pores in three alloy powders are comprehensively characterized and compared by SEM and CT. It is found that particle size, rotation speed of the electrode bar and alloy type all play a key role on pore characteristics. The following conclusions can be drawn from this study.

1. For a given alloy, pores are more likely to form in coarse particles, and the volume of pores increases with increasing particle size.
2. For a given alloy, the quantity percentage of powder with pores in the as-PREPed powder increases gradually with decreasing rotation speed.
3. The surface tension(γ) and density(ρ) of alloy play a key role in pore characteristics. The alloys with high γ/ρ have a large average particle size, which may cause more powders with pores. In addition, alloys with low surface tension are more likely to absorb argon. The quantity percentage of powder with pores in Ti-6Al-4V is the largest among three alloys.

## Data Availability

All data are included in the paper.
